# Bridging heat-flow and guarded-heater methods for thermoelectric module efficiency evaluation

**DOI:** 10.1016/j.isci.2026.115271

**Published:** 2026-03-07

**Authors:** Yasutaka Amagai, Kenjiro Okawa, Ryoji Funahashi, Atsushi Yamamoto, Michihiro Ohta

**Affiliations:** 1 National Institute of Advanced Industrial Science and Technology (AIST), National Metrology Institute of Japan (NMIJ), 1-1-1 Umezono, Tsukuba, Ibaraki 305-8563, Japan; 2 National Institute of Advanced Industrial Science and Technology (AIST), Global Research and Development Center for Business by Quantum-AI technology (G-QuAT), 1-1-1 Umezono, Tsukuba, Ibaraki 305-8560, Japan; 3 National Institute of Advanced Industrial Science and Technology (AIST), Nanomaterials Research Institute, 1-8-31 Midorigaoka, Ikeda, Osaka 563-8577, Japan; 4 National Institute of Advanced Industrial Science and Technology (AIST), Global Zero Emission Research Center (GZR), 16-1 Onogawa, Tsukuba, Ibaraki 305-8569, Japan

**Keywords:** Electrical engineering, Energy engineering, Physics

## Abstract

Reliable efficiency evaluations are recognized as one of the major challenges in thermoelectric technology. Existing heat-flow and guarded-heater methods suffer from uncertainties due to heat losses from the lateral surfaces of the thermoelectric module. Here, we introduce an apparatus that encloses the module with a thermally matched guard ring, separated by a vacuum gap, establishing near-isothermal sidewalls that suppress radiative losses from the lateral surfaces. Measurements with the hot side up to 703 K and an input heat flow up to 124 W showed input-output agreement within 0.5 W. Conversely, the unguarded configurations exhibited discrepancies exceeding 5 W. Thermal simulations qualitatively supported these trends, and a comparison with a reference instrument confirmed the electrical consistency while revealing the differences in thermal boundary conditions. This measurement configuration connects the two approaches, enabling reproducible efficiency evaluations with an expanded uncertainty of 1.4% and providing a technical basis that may inform future international standardization.

## Highlights

• Guard ring suppresses lateral heat leakage in TEMs
• Thermal-resistance matching ensures energy consistency
• Axial/radial flux ratio confirms quasi-1D heat flow
• 1.4% expanded uncertainty under high-temperature operation for oxide TEMs

## Graphical abstract

**Figure:**
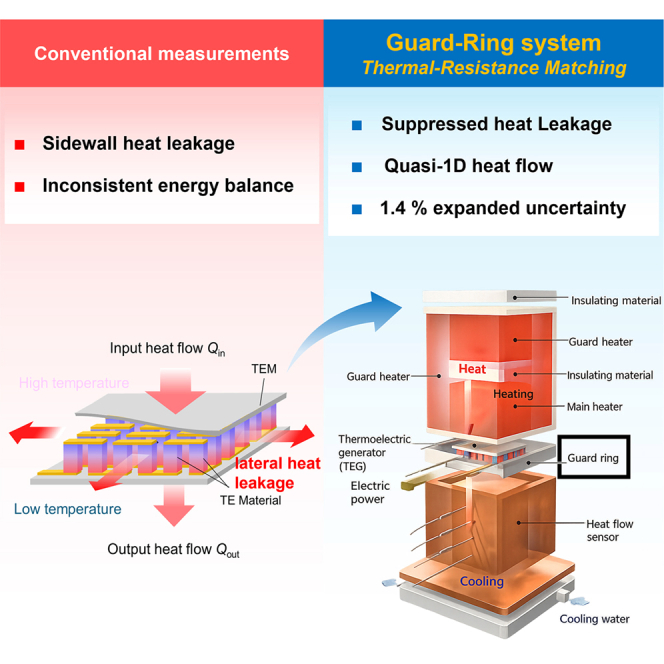

## Introduction

Thermoelectric modules (TEMs) have been considered as promising candidates for waste heat recovery and mobile power generation.^1^^,^^2^^,^^3^^,^^4^ Several reviews have emphasized that reliable thermoelectric measurements remain the cornerstone of connecting material research and device implementation.^5^^,^^6^^,^^7^^,^^8^ Recent advances in high-performance thermoelectric materials and module designs have further increased the demand for reproducible and reliable efficiency evaluation under realistic operating conditions. However, reliable evaluation of their efficiency remains challenging owing to limitations in existing measurement techniques. Conventional calorimetric approaches, including the heat-flow and guarded-heater method, often suffer from uncertainties arising from interfacial thermal resistance, convective instability, and, most critically, lateral-surface heat losses by radiative heat exchanges at elevated temperatures. These limitations have contributed to inconsistencies among laboratories, as documented in international round-robin comparisons: not only material-level property measurements^9^^,^^10^^,^^11^ but also more recent studies on TEMs themselves have revealed significant discrepancies in efficiency and heat-flow measurements.^12^^,^^13^^,^^14^^,^^15^ Such module-level inconsistencies underscore the urgent need for standardized methods. Best-practice guidelines emphasize measurement traceability, artifact suppression, and reproducibility.^16^

Over the past decades, several approaches have been proposed for evaluating module efficiency. Early studies introduced the Harman method in the 1950s,^17^ which was later refined for module-level figure-of-merit determination.^18^^,^^19^ Subsequent efforts established calorimetric guarded-heaters and heat-flow methods.^20^^,^^21^^,^^22^^,^^23^^,^^24^^,^^25^^,^^26^^,^^27^ Although these approaches have facilitated significant advances, they still rely primarily on post-measurement estimations of lateral-surface heat losses rather than their physical suppression. Recently, Kanno et al. demonstrated quantitatively that premature data acquisition and radiative side losses can introduce serious errors.^28^ More comprehensive theoretical modeling studies have further underscored the strong influence of lateral-surface heat transfer on module efficiency.^29^

Nevertheless, a physically engineered solution specifically designed for TEM efficiency measurements has remained elusive. In thermal conductivity measurements, for example, the guarded hot-plate method can employ homogeneous guard materials that surround the specimen and establish isothermal boundaries, which is now codified in ISO 8302 and ASTM C177.^30^^,^^31^ In contrast, TEMs are functional, heterogeneous multilayer devices incorporating electrodes and *p*-*n* legs, For this reason, surrounding the specimen with identical materials or measuring only a portion of the device is impractical. As a result, lateral heat loss in TEM measurements cannot be addressed using homogeneous guard concepts without introducing additional artifacts.

An alternative approach is to surround the module with thermal insulation; however, insulation-based suppression does not fully define or control lateral heat flow quantitatively and, therefore, relies on an implicit assumption of one-dimensionality. Moreover, the effectiveness of insulation depends sensitively on how the thermal insulation is applied, such as its thickness, coverage, and mechanical contact, which may vary between measurements and lead to limited reproducibility. This limitation becomes particularly critical at elevated temperatures, where lateral radiative losses can significantly perturb the energy balance. Under these constraints, a different guarding strategy is required for reliable TEM efficiency measurements.

Here, we present a measurement configuration that addresses lateral heat loss in TEM efficiency characterization. By enclosing the module with a vacuum-isolated thermal-impedance-matched guard ring, we established near-isothermal lateral surfaces that suppressed radiative exchange and promoted one-dimensional heat flow. The purpose of this study is to demonstrate an instrumentation and measurement configuration for improving energy consistency in TEM efficiency evaluation, rather than to establish a formal measurement standard.

## Results

### Design and implementation of the guard-ring apparatus

**Figure 1:**
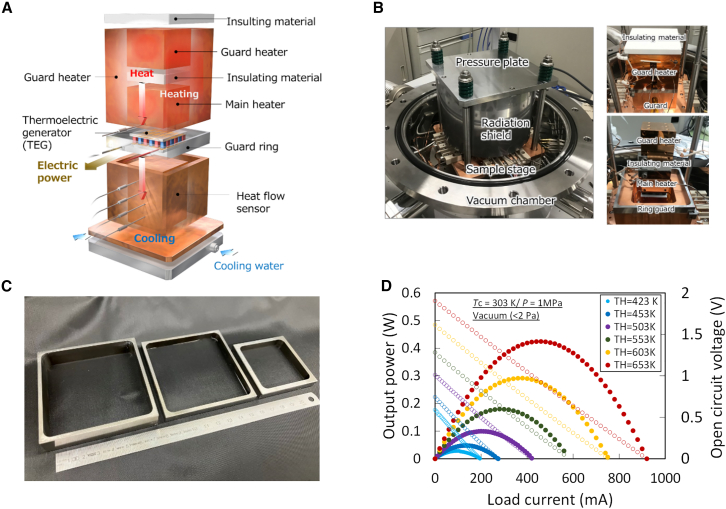
Guard-ring-based developed apparatus for TEM efficiency evaluation (A) Schematic of the measurement setup. The TEM is sandwiched between hot and cold copper blocks and laterally enclosed by a thermally matched guard ring separated by a narrow vacuum gap. Guard heaters, insulating materials, and a water-cooled stage ensure controlled boundary conditions for one-dimensional heat flow. (B) Inside view of the vacuum chamber showing the pressure plate, outer radiation shield, and guard structure. The insets highlight the arrangement of the guard heater, main heater, insulating layers, and guard ring surrounding the TEM. (C) Image of the guard ring. The guard ring is thermally coupled to the hot stage via a graphite sheet and to the cold stage via a thin silicone grease layer, as indicated, ensuring stable mechanical and thermal contact. (D) Representative electrical output characteristics of a TEM measured with the developed system under vacuum. The output power and open-circuit voltage are shown as functions of load current at different hot-side temperatures (cold side fixed at 303 K).

To suppress the lateral radiative heat loss and ensure an accurate evaluation of the TEM efficiency, we developed a custom guard-ring-based measurement apparatus (Figures 1A and 1B). Oxide-based TEMs^30^^,^^31^^,^^32^ were used in this study to validate the apparatus performance (see STAR Methods for details). We note that similar principles have been applied in guarded hot-plate metrology to suppress lateral losses in thermal conductivity standards,^30^^,^^31^ and related guard-heater concepts have been explored in TEM testing.^33^ Our approach extends this design philosophy by introducing a passive impedance-matched guard specifically tailored for the evaluation of TEM efficiency.

The apparatus was designed to promote quasi-one-dimensional heat flow along the axial direction of the module by minimizing radiative exchange from the lateral surface of the TEM. This radiative heat exchange from the lateral surface becomes a dominant loss channel under high-temperature and vacuum conditions. The TEM was sandwiched between high- and low-temperature copper blocks and laterally enclosed by a stainless-steel guard ring, forming a square cross-section (Figure 1C). A vacuum gap of approximately 10 mm separated the TEM from the guard, acting as a thermal barrier for conduction while allowing radiative interaction. The key design principle was to match the lateral temperature of the guard to that of the TEM side surfaces, thereby eliminating the net radiative heat exchange. This thermal equilibrium was achieved by tuning the guard thickness to balance the thermal impedance with the axial heat flow through the module. In practice, the guard ring was fabricated with the same height as the TEM to ensure comparable thermal boundary conditions, and its thickness was determined based on the estimated thermal conductance of the module, such that the lateral temperature profile of the guard tracked that of the module sidewalls.

From the viewpoint of a thermal equivalent circuit, the system can be approximated as a thermal Wheatstone bridge, wherein the heat-flow balance condition is achieved when the lateral thermal resistance of the guard matches the effective axial resistance of the TEM. To evaluate the output characteristics of the TEM, the power curve was measured under controlled conditions using a programmable electronic load, which enabled continuous current sweeping and simultaneous voltage recording. A representative power curve obtained using this setup is shown in Figure 1D.

**Figure 2:**
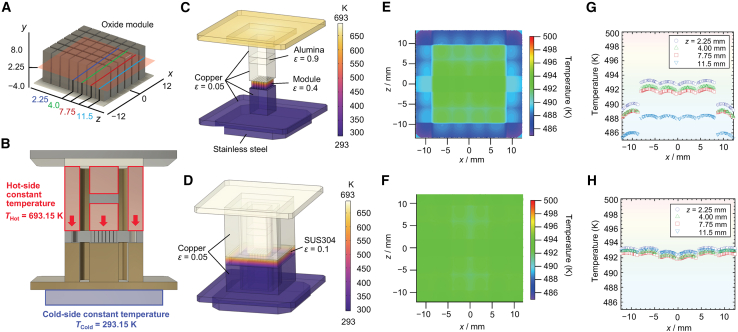
Finite element simulations of lateral radiative heat loss and suppression by the guard-ring design (A) Simulation model of the TEM, copper hot/cold blocks, and guard ring, showing the locations (*z* = 2.25, 4.00, 7.75, and 11.5 mm) where radial temperature profiles were extracted. (B) The depiction of the thermal boundary condition using this analysis. (C and D) Temperature distributions of the TEM under vacuum conditions: (C) without the guard ring, strong lateral gradients develop along the sidewalls; (D) with the guard ring, the lateral boundary becomes nearly isothermal. The materials and their emissivity values used in this analysis are also shown. (E and F) Top-view temperature maps of the oxide module obtained from FEM simulation: (E) without the guard ring, pronounced edge cooling owing to lateral radiation is visible; (F) with the guard ring, the lateral surfaces are thermally equilibrated and the temperature distribution across the top surface becomes homogenized. (G and H) Radial temperature profiles of the six pairs of legs (Figure 2A), extracted along four leg rows from the module edge toward the center (positions at *z* = 2.25, 4.00, 7.75, and 11.5 mm): (G) without the guard ring, a pronounced global concave curvature (center hot, edge cool) is observed across the module; (H) with the guard ring, the global curvature is strongly suppressed and the lateral distribution becomes homogenized; however, a local concave profile within individual legs remains owing to intrinsic lateral heat spreading.

The simulation domain included the TEM, hot and cold copper blocks, and the surrounding guard-ring structure (Figure 2A; see STAR Methods for details). The finite element analysis was used to visualize heat-flow distributions and to qualitatively assess the effectiveness of the guard-ring configuration in suppressing lateral heat leakage. Without the guard (Figures 2C, 2E, and 2G), lateral gradients developed across the sidewalls, resulting in outward radiative losses and a pronounced global concave curvature in the radial temperature distribution. With the guard in place (Figures 2D, 2F, and 2H), the temperature closely followed that of the adjacent TEM surfaces, producing a near-isothermal lateral boundary condition. Consequently, the edge cooling of the TEM was significantly reduced, and the overall lateral temperature distribution became more homogeneous. For instance, at *z* = 2.25 mm, without the guard in place, the temperature at the module edge (*x* = ±12 mm) was about 490 K, whereas at a radial position 5 mm inward from the edge (*x* = ±7 mm), it was about 493 K, indicating a temperature drop of approximately 3 K (Figure 2E). With the guard in place, by contrast, both edge and center remained nearly 493 K, demonstrating the homogenization of the lateral profile (Figure 2F). The radial temperature profiles of the six legs extracted along the four leg rows from the module edge toward the center (Figures 2G and 2H) clearly confirmed that the guard effectively suppressed the global concave curvature of the module. A local concave profile (center-hot, edge-cool) within the individual legs remained visible owing to the intrinsic lateral heat spreading.

In this sense, the simulation results indicate that the guard ring equalizes the macroscopic outer boundary condition, thereby suppressing radiation-induced edge cooling, such that the overall temperature field approaches a nearly one-dimensional distribution. Although local concavity persists at the individual leg level, the macroscopic heat flow across the module effectively becomes quasi-one-dimensional. The quantitative validation of one-dimensional heat flow relevant to efficiency evaluation is provided by experimental energy-balance measurements and uncertainty analysis presented in the following sections.

### Experimental validation of heat loss suppression

**Figure 3:**
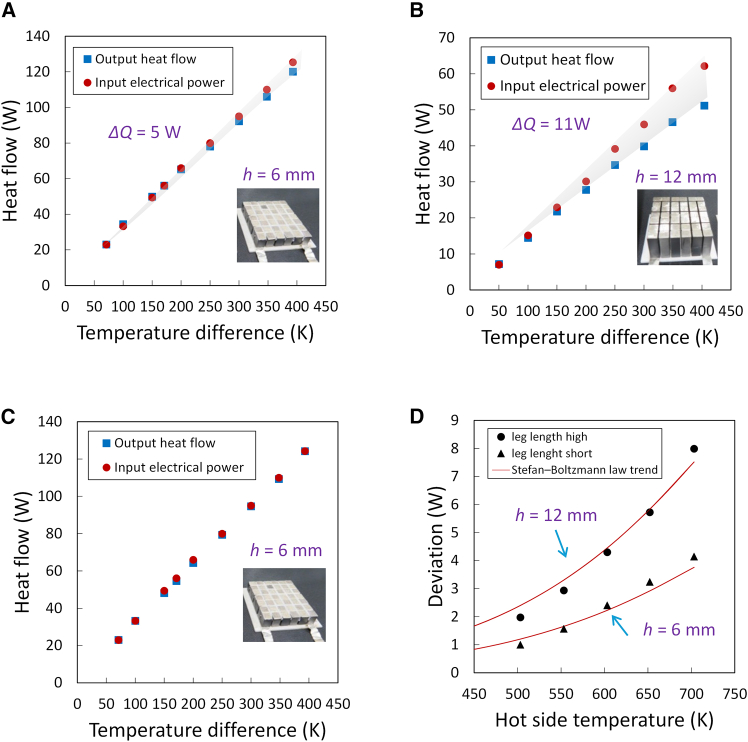
Comparison of heat flow performance under various structural and thermal guard conditions (A) Heat input and output characteristics of a TEM with a 6 mm leg length measured without a thermal guard. (B) Same measurement for a module with a longer 12 mm leg length, exhibiting increased radiative loss. (C) Performance of the 6-mm module with a thermal guard, demonstrating improved input–output agreement. (D) Illustrative example highlighting deviation in heat flow caused by radiative heat exchanges as a function of hot-side temperature, highlighting the dependence on leg length.

To quantitatively assess the effectiveness of the guard-ring structure in suppressing the lateral radiative losses, we conducted a series of heat flow measurements with and without the guard ring under identical experimental conditions. The input heat flow (*Q*_in_) was calculated from the electrical power supplied to the main heater, which was determined from the direct current (DC) current and voltage. The output heat flow (*Q*_out_) was estimated from the heat flow sensor on the cold side. Specifically, *Q*_out_ was obtained from the measured temperature gradient (Δ*T*) across a calibrated copper block with known thermal conductivity (*k*), cross-sectional area (*A*), and thickness (*L*), according to Fourier ’s law: *Q*_out_ = (*kA*Δ*T*)/*L*. In the absence of the guard ring, a significant discrepancy between *Q*_in_ and *Q*_out_ was observed, particularly at elevated temperatures (Figure 3A). For the oxide module with a height of 6 mm, the difference between the input and output heat flows exceeded 5 W when the hot-side temperature was increased to approximately 400 K and the cold side was maintained at 303 K. This deviation reflects substantial heat losses from the lateral surfaces of the module, which become increasingly severe at higher temperatures owing to radiative effects. The effect of radiative heat loss was more pronounced for longer legs because of their larger surface area (Figure 3B). When the guard ring was applied, the discrepancy between *Q*_in_ and *Q*_out_ was substantially reduced to less than 0.5 W across the entire temperature range tested (*T*_h_ = 353–703 K) (Figure 3C). This corresponds to an energy-balance agreement better than 0.5%, demonstrating that the guard ring effectively suppresses the dominant mode of heat loss under vacuum, sidewall radiation. The standard deviation obtained from repeated measurements was smaller than the symbol size in the corresponding plots and is, therefore, not visually discernible; its contribution is included in the type A component of the Guide to the Expression of Uncertainty in Measurement (GUM)-compliant uncertainty analysis.^34^

To gain physical insight into the lateral thermal loss mechanism, we examined the heat flow difference (*Q*_in_ − *Q*_out_) as a function of the hot-side temperature for oxide modules with leg lengths of 6 and 12 mm (Figure 3D). The temperature dependence of the heat-flow imbalance qualitatively follows the trend expected from radiative heat transfer, as described by the Stefan-Boltzmann law. Here, the observed trend is used to illustrate the increasing importance of lateral radiative losses at elevated temperatures, rather than to establish a quantitative radiative scaling. The application of the guard ring significantly reduced the magnitude of the heat-flow imbalance and suppressed its temperature dependence, indicating that the lateral temperature equilibrium imposed by the guard structure effectively mitigates radiation-driven edge cooling. This result demonstrates that thermal-impedance matching of lateral surfaces is an effective strategy for reducing non-axial heat transfer. Importantly, the conclusions regarding measurement accuracy and energy consistency are derived from experimental energy-balance measurements and uncertainty analysis, and do not rely on detailed radiative finite element method (FEM) modeling.

### Comparison with reference instrument

To assess the consistency of the guard-ring apparatus with established TEM evaluation practices, we performed a cross-comparison with a reference instrument (PEM2, ADVANCE RIKO, Inc., Japan) that has been widely used for TEM characterization, including international round-robin studies.^10^

**Figure 4:**
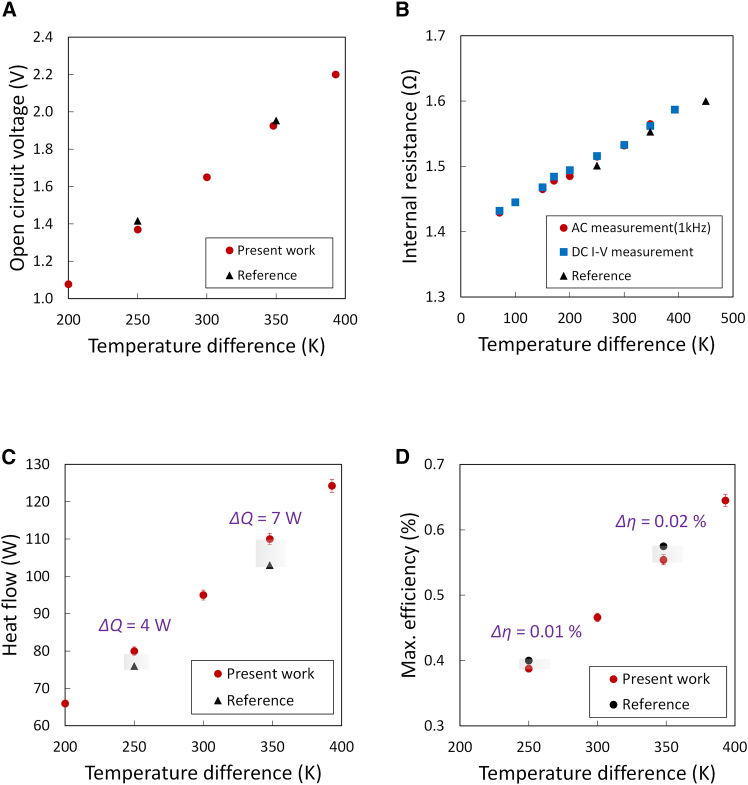
Comparison of the guard-ring apparatus with commercial precision instruments (A) Open-circuit voltage as a function of Δ*T*. Excellent agreement is observed between the present work (red circles) and the reference system (black triangles), confirming the validity of the electrical measurement pathway. (B) Internal resistance determined by AC (red circles) and DC *I*–*V* measurements (blue squares) using the guard-ring apparatus, compared with the reference system (black triangles). The results are consistent within 0.1%, further validating the electrical characterization. (C) Heat flow versus temperature difference across the oxide module (Δ*T*). The results obtained with the present guard-ring apparatus (red circles) are compared with those from a commercial reference calorimetric system (black triangles, PEM2). The present system shows consistent values, whereas deviations in the reference are attributed to radiative heat losses. (D) Maximum efficiency as a function of Δ*T*. Although the electrical parameters show strong agreement, systematic deviations appear in the efficiency values. This behavior is consistent with differences in lateral heat-loss treatment between the two measurement configurations. The guard-ring apparatus physically suppresses lateral radiative exchange, enabling internally consistent efficiency evaluation under the defined boundary conditions.

PEM2 evaluates efficiency by supplying electrical power to a heater attached to the hot side, measuring the heat flux conducted to the cold side with a built-in thermoelectric heat-flow sensor, and then defining the efficiency as the ratio of electrical output power to input heat flow. The instrument incorporates radiation shields and operates under reduced pressure conditions to minimize convective and radiative losses. However, these measures do not establish a fully isothermal lateral boundary; residual sidewall radiation remains, requiring either implicit treatment or correction. By contrast, the guard-ring apparatus enforces thermal equilibrium at the lateral surfaces via thermal impedance matching, thereby physically suppressing radiative losses rather than relying solely on shielding or estimation. Figures 4A–4D compare the open-circuit voltage, internal electrical resistance, heat flow, and maximum efficiency values obtained with the guard-ring system and PEM2.

The reported resistance values include not only the thermoelectric legs and electrodes but also the silver lead wires attached to the module. Therefore, the internal resistance was defined between the outermost ends of the lead wires. The open-circuit voltage (*V*_oc_) and internal resistance (*R*) measured with the guard-ring apparatus using the steady-state DC *I*-*V* and AC four-probe methods closely matched those obtained with PEM2 (Figures 4A and 4B), with differences below 0.1%. This agreement between AC and DC methods is further supported by the fact that the oxide-based TEMs exhibit relatively small Peltier-induced fluctuations, minimizing the difference between AC and DC resistance measurements. The internal electrical resistance included the resistance of the electric lead made of silver, suggesting that the uncertainty contribution from the resistance measurement was negligible compared with those of the calorimetric balance and temperature difference terms.

In contrast to the electrical properties, differences were observed in the thermal evaluation results. While the overall trends were similar, PEM2 yielded slightly lower heat-flow values at elevated temperatures (Figure 4C). The magnitude of this deviation exceeded the relative expanded uncertainty^34^ of the guard-ring measurement (1.4%, *k* = 2), where *k* is a coverage factor indicating a 95% level of confidence (see STAR Methods and supplemental information for details). A detailed uncertainty analysis is provided in supplemental information as Tables S1–S3. Furthermore, the relative contributions of the dominant uncertainty sources—input heat flow (*Q*_in_), temperature difference (Δ*T*), and electrical output power (*P*), including residual sidewall radiation effects—are explicitly summarized in Table S4. Although the uncertainty specification of PEM2 is not publicly available, which prevents a fully quantitative comparison, the systematic negative bias observed in this study is consistent with the residual lateral radiative losses inherent to its measurement principle. When conversion efficiency was evaluated, systematic deviations were evident (Figure 4D). PEM2 exhibited higher efficiency values across the tested temperature range, which is consistent with the effect of lateral radiation losses not being explicitly suppressed. These systematic deviations are greater than the estimated uncertainty of 1.4%, again supporting the interpretation that they originate from differences in the thermal boundary conditions rather than from measurement scatter. By equalizing the boundary conditions and suppressing radiative exchange, the guard-ring apparatus provided efficiency values that were closer to the intrinsic device performance.

Collectively, these results demonstrate that the guard-ring design achieves the electrical characterization consistent with established standard instruments, while highlighting the impact of lateral thermal boundary conditions on calorimetric efficiency evaluation. This comparison is presented as a qualitative consistency check rather than as a quantitative benchmark of measurement accuracy.

## Discussion

The guard-ring configuration presented in this study should be understood as one practical implementation of the proposed thermal-resistance-matching design principle, rather than as the principle itself. A comparison with a widely used commercial precision instrument demonstrates consistency in electrical characterization and highlights differences in thermal boundary conditions between the two measurement configurations. The system reproduces the electrical characterization being consistent with commercial precision instruments while revealing their thermal boundary effects associated with their measurement configurations. This dual capability improves laboratory measurement consistency and illustrates a possible pathway toward harmonizing TEM characterization practices. From a methodological perspective, the present approach can be viewed as complementary to existing ISO- and ASTM-related calorimetric concepts, in that it addresses lateral heat-loss effects that are not explicitly controlled in standard guarded-heater or heat-flow configurations.

By physically suppressing radiation-induced artifacts instead of relying on post-measurement correction procedures, the guard-ring design offers a practical path toward a reproducible and traceable efficiency evaluation. Radiative losses, which increase strongly with hot-side temperature as expected from radiative heat-transfer principles, were observed in unguarded setups. Unlike blackbody-based maximal radiation loss as a post-measurement bound and FEM-based studies that highlight radiative bypass as a source of interlaboratory discrepancies, our approach provides an experimental means to physically suppress these effects rather than estimate them. Furthermore, the apparatus distinguished intrinsic TEM behavior from extrinsic artifacts; discrepancies increased with module height in unguarded cases, but vanished when the guard was applied, validating that lateral heat loss can be geometrically controlled. This observation aligns with prior theoretical analyses, indicating that incorporating structures to mitigate side-surface radiation can significantly enhance the conversion efficiency.^26^ Our study provides an experimental validation of this concept by demonstrating that a guard ring physically enforces such suppression.

A long-standing issue in the thermoelectric metrology community concerns the systematic biases of different calorimetric approaches.^21^^,^^22^^,^^23^ Recent work by Ziolkowski et al.^35^ systematically compared reference-based and absolute heat-flow techniques, demonstrating that guard-heater methods inherently underestimate efficiency due to sidewall radiation, whereas heat-flow calorimetry tends to overestimate it. Their analysis underscored that absolute heat-flow measurement is essential for standardization, highlighting the biases that our guard-ring apparatus experimentally resolves. Guard-heater methods generally underestimate efficiency, because the heater power supplied to maintain the guard temperature includes residual sidewall radiation that does not contribute to useful conversion, thereby underestimating the efficiency. Heat-flow calorimetry often overestimates efficiency instead, because it records only the transmitted heat flux *Q*_out_ at the cold side while maintaining the average Δ*T* across the module; this makes the denominator in the efficiency calculation artificially small. In practice, sidewall radiation also perturbs the lateral temperature distribution of the legs, reducing the contribution of edge elements to the total Seebeck voltage. Under certain conditions, this reduction in *P*_out_ may partially offset the smaller *Q*_out_, leading to apparent cancellation and efficiencies that seem nearly unchanged. Such variability, which depends strongly on geometry, material system, and measurement conditions, provide one possible explanation on why interlaboratory comparisons have repeatedly yielded inconsistent results. These considerations illustrate why different calorimetric approaches can yield systematically different efficiency values under certain measurement conditions.

A further conceptual issue concerns which heat flow should be taken as the basis for efficiency definition. According to the rigorous thermodynamic definition, efficiency should be referenced to the total input heat *Q*_in_ supplied to the module, including sidewall losses. This viewpoint aligns with practical interests as well, since system designers and end users of TEMs ultimately care about the fraction of the supplied heat from the heat source that is converted to electricity. In practice, however, directly quantifying *Q*_in_ from heaters or furnaces is rarely feasible, leading most laboratory studies to use *Q*_out_ as a surrogate. Moreover, radiative exchange with the surroundings at elevated temperatures makes accurate and repeatable measurement even more challenging. Consequently, this mismatch between theoretical definition and measurement convenience has contributed to the ambiguity in reported efficiencies.

Taken together, the present guard-ring apparatus mitigates this problem by physically suppressing sidewall losses, thereby ensuring that *Q*_in_ is nearly equal to *Q*_out_. As a result, the measured efficiency becomes insensitive to whether it is referenced to *Q*_in_ or *Q*_out_, aligning experimental practice with thermodynamic rigor and providing an experimental path toward bridging different calorimetric definitions.

The key insight provided by this study is that physically matching the lateral thermal resistance of a TEM stabilizes the energy balance during efficiency evaluation by suppressing non-axial heat losses. All supporting analyses, including numerical simulations, radiative considerations, and comparative measurements, serve to illustrate this effect and do not constitute independent performance claims. Beyond laboratory measurements, the proposed configuration provides a practical framework for improving the reproducibility of TEM efficiency evaluation, which may inform future international standardization efforts.

### Limitations of the study

Despite its significant contribution to harmonizing thermoelectric metrology, this study had several technical limitations and challenges that should be addressed in future work. The thermal impedance of the guard must be tuned to the module thermal resistance, which requires prior knowledge of the device parameters, and the guard height must match each sample. In addition, this approach balances lateral exchange in one direction but does not fully eliminate the radial nature of radiation. These design constraints do not fundamentally limit the general applicability of the guard-ring concept, but rather define practical parameters that can be adjusted for different module geometries.

Furthermore, the present validation was limited to oxide-based TEMs with relatively large leg dimensions. Although these modules are chemically stable and well suited for fidelity testing and possible candidate for standard modules, their relatively low efficiency may not fully represent the behavior of state-of-the-art Bi_2_Te_3_ and half-Heusler-based module. Additional investigations are required to confirm whether the guard-ring concept remains effective for high-performance materials with stronger Peltier effects. Another type of module we should address is the metal-based module having small thermal and electrical resistances.^36^ Addressing these issues will be critical for extending the generality of this approach.

## Resource availability

### Lead contact

Requests for further information and resources should be directed to and will be fulfilled by the lead contact, Yasutaka Amagai (y-amagai@aist.go.jp).

### Materials availability

This study did not generate new unique materials. Oxide-based TEM samples were fabricated in-house as described in STAR Methods section.

### Data and code availability

The data supporting the findings of this study are available from the corresponding authors upon request. This paper does not report original code. Any additional information required to reanalyze the data reported in this paper is available from the lead contact upon request.

## Acknowledgments

The authors are grateful to H. Takazawa and H. Obara of AIST for their contributions to the initial apparatus design carried out at AIST, which served as the basis for the present study, and to K. K. Johari of AIST for the performance evaluation of the oxide module with the PEM-2 system. They are also grateful to M. Akoshima of AIST for her valuable insight into the guard ring design at the early stages of apparatus design and providing the reference material. The authors acknowledge N.-H. Kanko, N. Sakamoto, and M. Maruyama of AIST for their fruitful discussions regarding the interpretation of the numerical calculations. Finally, we would like to thank P. Ziolkowski and E. Muller of DLR in Germany for valuable discussions following our preliminary online presentation of this work. Their comments and questions on the apparatus structure were highly insightful to further refine the apparatus. They also acknowledge their contributions to ongoing international comparison activities, which we believe will remain an important cornerstone to future standardization. This work was supported by the 10.13039/501100001863New Energy and Industrial Technology Development Organization (NEDO) under the Research and Development Program for Promoting Innovative Clean Energy Technologies Through International Collaboration (project no. JPNP20005, FY2020–FY2023).

## Author contributions

M.O. and A.Y. jointly conceived the project on TEM evaluation and advanced international interlaboratory comparisons. Y.A. and K.O. developed the apparatus and conceived the thermally matched ring-guard design. R.F. designed, fabricated, and provided oxide TEMs with different leg lengths. K.O. performed the numerical simulations. Y.A. performed the precision measurements, data analysis, and measurement of uncertainty. Y.A. prepared the initial draft of the manuscript. All the authors discussed the results and contributed to the final version of the manuscript.

## Declaration of interests

All authors declare the following competing interest: they are inventors on Japanese Patent Application No. 2022-142726 (filed 2022/09/08, published as Japanese Patent Laid-Open Publication No. 2024-038604, currently under examination as of March 21, 2024) related to thermoelectric module efficiency evaluation methods.

## STAR★Methods

### Key resources table

**Table:** 

REAGENT or RESOURCE	SOURCE	IDENTIFIER
Custom-built oxide-based thermoelectric module	This paper	–
Flexible graphite sheet (GraFoil®, TG-411, 0.38 mm)	NeoGraf Co., Ltd.	Product: GraFoil® TG-411
Thermal interface grease (KS-609)	Shin-Etsu Chemical Co., Ltd.	Product: KS-609
Electronic load instrument (PLZ164WA)	KIKUSUI Electronics Co., Ltd.	Model: PLZ164WA
DC power supply to the heater (PAN160-7A)	KIKUSUI Electronics Co., Ltd.	Model: PAN160-7A
DC-inverter chiller (RKE1500)	ORION Machinery Co., Ltd.	Model: RKE1500
Switch/Multimeter system (3706A)	Keithley Instruments	Model: 3706A
Standard temperature device Zero-con® (ZC-114A)	Coper Electronics Co., Ltd.	Model: ZC-114A
Calibrated reference ceramic block (AL1, polycrystalline Al_2_O_3_)	Japan Fine Ceramics Center (JFCC)	AL1; see Ref.^37^
Bench top water circulator (CB100)	Yamato Scientific Co., Ltd.	Model: CB100
Custom-built vacuum chamber	This paper	–
Custom-built heat flow sensor	This paper	–
Vacuum pump	Pfeiffer Vacuum Co., Ltd.	Model: HiCUbe80 Classic

### Experimental model and study participant details

Omitted as our study does not involve biological models.

### Method details

#### Thermoelectric modules (TEMs)

The TEMs evaluated in this study were based on oxide materials, which were selected for their chemical stability and reliable operation at elevated temperatures.^30^ Each module comprised 21 *p*–*n* legs fabricated from *p*-type Ca_2.7_Bi_0.3_Co_4_O_9_ and *n*-type CaMn_0.98_Mo_0.02_O_3_, with device dimensions of 30 mm × 30 mm × 6 mm and 30 mm × 30 mm × 12 mm. Each leg has a cross section of 3.5 mm × 3.5 mm. Aluminum oxide and silver were used as the substrate and electrode, respectively. In addition, compared with the high-performance Bi_2_Te_3_-based modules, oxide modules offer superior chemical and thermal stability at high temperatures and exhibit relatively smaller Peltier-induced variations, making them particularly suitable for evaluating measurement fidelity. While they are not intended to represent cutting-edge thermoelectric performance, their robustness provides a reliable platform for testing the accuracy of efficiency evaluation methodologies.

#### Guard-ring apparatus design details and measurement procedure

The guard ring was mechanically fixed to the hot and cold stages. On the hot side, a thin flexible graphite sheet (NeoGraf co., Ltd., GraFoil®, TG-411, thickness 0.38 mm) was inserted to improve thermal contact and accommodate thermal expansion, whereas on the cold side, a thin layer of silicone-based thermal interface grease(Shin-Etsu chemical co., Ltd., thermal interface grease KS-609) was applied. These interfaces ensured a reliable thermal connection of the guard to the respective heat sink and outer guard while minimizing the interfacial resistance. A compressive load of approximately 1 MPa was applied via a spring-loaded stage and closely monitored to minimize interfacial thermal resistance. The same graphite sheets on the hot side and silicone oil compound on the cold side were used to improve contact. The open-circuit voltage was measured to confirm sufficient compressive load. As the compressive load increased, the open-circuit voltage increased and stabilized when the compressive load exceeded 1 MPa, which can be attributed to reduced thermal contact resistance between the module and stage, leading to a larger temperature difference across the module. The electrical load on the module was controlled by an electronic load device (KIKUSUI electronics co., Ltd., electronic load, PLZ164WA ), which allowed continuous variation of the external resistance during measurement.

Heating was provided by a resistive main heater and auxiliary guard heaters surrounding the main heater with a stable DC power supply(KIKUSUI electronics co., Ltd., DC-power supply, PAN160-7A). The cold side was stabilized using a closed-loop chiller with ±0.1 K stability (ORION machinery co., Ltd., DC-inverter chiller, RKE1500). The temperatures were monitored by T- and R-type thermocouples, and the output voltage from these thermocouples was measured using a DC voltmeter with a multiplexer system(Keithley Instruments, switch/multimeter, 3706A). A commercially available reference bath equipped with a Peltier cooling device was employed to ensure that the cold junction remained at 273 ± 0.02 K for extended durations by automatically maintaining a mixture of ice and water (Coper electronics co., Ltd., standard temperature device Zero-con®, ZC-114A). The R- and T-type thermocouples measured the hot and cold sides of the module, respectively. Small holes were drilled in the copper heater and cold stages to insert the thermocouples approximately 1 mm above and below the module surfaces, enabling accurate measurement of the temperature difference across the module. The input heat was determined from the electrical current and voltage supplied to the cartridge heaters embedded in the copper block. The output heat flow was evaluated based on the measured temperature gradient across a calibrated copper block of known thermal diffusivity. (Japan Fine Ceramics Center, JFCC, AL1, Polycrystalline aluminum oxide).^37^ The thermal conductivity of this reference ceramic material was obtained by multiplying its thermal diffusivity by the specific heat capacity and density.

All the measurements were performed in a custom-built vacuum chamber, which was water-cooled with a water-chiller (Yamato scientific co., Ltd., Bench top water circulator, CB100) to suppress heating of the chamber walls and to minimize parasitic radiative exchange with the surroundings, and parasitic thermoelectric voltage induced in electrical leads and cables.

#### Numerical simulations

Numerical simulations were performed using COMSOL Multiphysics(version 6.0, COMSOL, Stockholm, Sweden). The Heat Transfer in Solids interface was coupled with the Surface-to-Surface Radiation interface. The governing equation for steady state heat conduction in the solid domain is:

(Equation 1) ∇・(–*κ*(*T*)∇*T*) = *Q*

where *κ*(*T*) is the temperature dependent thermal conductivity obtained from the COMSOL materials database, *T* is the temperature, and *Q* represents internal volumetric heat sources, if any.

Surface-to-Surface radiation between diffuse and gray surfaces was modeled using the radiosity method. The net radiative heat flux *q*_rad_ at a surface is defined as:

(Equation 2) *q*_rad_ = *ε*(*J* − *G*), *J* = *εσT*^4^ + (1-*ε*)*G*, *G* = Σ^N^_*j* = 1_*F*_*ij*_*J*_*j*_

where *ε* is the surface emissivity, *J* is the radiosity, and *G* is the incident irradiation: *J* = *εσT*^4^ + (1-*ε*)*G*, *G* = Σ^N^_*j* = 1_ *F*_*ij*_*J*_*j*_. Here, *σ* is the Stefan-Boltzmann constant and *F*_*ij*_ is the view factor between surfaces *I* and *j*.^38^

The finite element method (FEM) domain included the TEM, copper blocks, and guard ring. Boundary conditions were set as follows the main heater and guard heater were maintained at 693.15 K, the water-cooled section at the bottom was fixed at 293.15 K, and the lateral and bottom surfaces were thermally insulated except for radiative boundaries. The chamber was assumed to be under vacuum; therefore, convective heat transfer was neglected. Material properties (thermal conductivity, density, specific heat) were taken from the COMSOL material library; emissivity values were assigned based on representative literature values because direct measurement was impractical. The emissivity values were set to 0.9 for the oxide module, 0.05 for polished copper, and 0.4 for stainless steel (AISI 403, JIS SUS403). For the material properties, database values of the thermophysical property were used for polished copper, and stainless steel, while the oxide module properties were based on previously reported data.^30^ The simulated TEM comprised 21 pairs of *p*–*n* legs (*p*-type Ca_2_._7_Bi_0_._3_Co_4_O_9_ and *n*-type CaMn_0_._98_Mo_0_._02_O_3_), each leg having dimensions of 3.5 mm × 3.5 mm × 6 mm. The substrates were made of purity aluminum oxide with a thickness of 0.8 mm, and the electrodes were pure silver sheets with a thickness of 0.1 mm. A mesh independence check was performed using COMSOL’s finest physics-controlled mesh.

**Table:** Thermophysical parameters used in the FEM simulation.

Components	Materials	Heat capacity [J/(kg K)]	Density [kg/m^3^]	Thermal conductivity [W/(m K)]	Emissivity
Thermoelectric generator	Thermoelectric oxide materials	154	7700	*κ*(*T*)^a^	0.4
Main heater / Guarded heater	Copper	385	8940	400	0.5
Insulating materials	Alumina	900	3900	27	0.9
Guard ring	Stainless Steel Annealed SUS 403	500	7930	16.4	0.1
Support parts	Steel AlSl 4340	475	7850	44.5	0.2

*κ*(*T*)^a^ = 1.6 (300 K), 1.6 (350 K), 1.75 (400 K).

### Quantification and statistical analysis

Uncertainty evaluation was conducted in accordance with the *Guide to the Expression of Uncertainty in Measurement* (GUM, JCGM 100:2008).^34^ We combined Type-A and Type-B components by root-sum-of-squares and computed the effective degrees of freedom using the Welch–Satterthwaite formula.

#### Main contributions

• Heat input measurement (voltage/current stability; Type-A with five repeats per condition, *n* = 5).
• Lateral heat loss from the TEM side surface (Type-B; modeled from bounding analysis of radiative exchange under guard/no-guard conditions).
• Thermocouple positioning for obtaining Δ*T* across the TEM (Type-B; placement tolerance converted from a rectangular bound to a standard uncertainty).
• Electrical power measurement (negligible compared with heat-flow or temperature measurement).

For Type-A terms we used the freedom as *n*-1. Type-B terms were treated with infinity. Consequently, the combined standard uncertainty and effective degrees of freedom was evaluated. Across all operating conditions, the calculated effective degree of freedom is so large that the coverage factor for 95 % confidence level is effectively normal value (*k* =1.96). We therefore report a rounded *k* = 2. Expanded uncertainty of the efficiency evaluate is 1.4% (*k* = 2, 95% confidence level). All electrical and thermal measurement instruments used in this study were calibrated against traceable standards prior to the measurements.

## Supplemental information

Document S1. Figures S1 and S2, Tables S1–S4, and Data/Methods S1
