# Supplementary material for: Bridging heat-flow and guarded-heater methods for thermoelectric module efficiency evaluation
Source: iScience. 2026 Mar 7;29(4):115271. doi: 10.1016/j.isci.2026.115271 (PMC13018900; doi:10.1016/j.isci.2026.115271)
Supplement: Document S1. Figures S1 and S2, Tables S1–S4, and Data/Methods S1 [file mmc1.pdf]

iScience, Volume *29*

Supplemental information

Bridging heat-flow and guarded-heater methods

for thermoelectric module efficiency evaluation

Yasutaka Amagai, Kenjiro Okawa, Ryoji Funahashi, Atsushi Yamamoto, and Michihiro Ohta

Data/Methods S1: Detailed uncertainty budget and supporting numerical simulations.

Uncertainty evaluation was conducted following the ISO GUM framework. Tables S1–S4 present the detailed uncertainty budgets. Additional FEM simulations supporting the discussion are shown in Figures S1 and S2. Thermophysical parameters correspond to the values used in the main text.

Table S1. Sub-budget for input heat flow Q_{in}

Source of uncertainty	Type	Statistical distribution	Sensitivity coefficient	Value	Standard uncertainty
Voltage (guarded heater)	B	Rectangular	6.0×10^{-5} (1/V)	1.0×10^{-5} (V)	3.5×10^{-10}
Current (guarded heater)	B	Rectangular	6.0×10^{-3} (1/A)	1.0×10^{-5} (A)	3.5×10^{-10}
Guard coupling (upper bound 0.1 W)	B	Rectangular	5.2×10^{-5} (1/W)	0.1 (W)	3.0×10^{-6}
Heat loss (side surface)	B	Rectangular	5.2×10^{-5} (1/W)	1 (W)	3.0×10^{-6}
Repeated measurement (current)	A	Normal	6.0×10^{-5} (1/V)	1.0×10^{-3} (V)	3.5×10^{-8}
Repeated measurement (voltage)	A	Normal	6.0×10^{-3} (1/A)	1.0×10^{-3} (A)	3.5×10^{-8}

Table S2. Sub-budget for influence of temperature difference ΔT on η

Source	Type	Statistical distribution	Sensitivity coefficient	Value	Standard uncertainty
R-thermocouple	B	Rectangular	1.8×10^{-5} (K)	1.5 (K)	2.7×10^{-5}
T-thermocouple	B	Rectangular	1.8×10^{-5} (K)	1.0 (K)	1.8×10^{-5}
Thermocouple misalignment	B	Rectangular	1.8×10^{-5} (K)	2 (K)	3.6×10^{-5}

Repeated measurement (temperature)	A	Normal	1.8×10^{-5} (K)	0.1 (K)	1.8×10^{-6}

Table S3. Sub-budget for electrical output *P*

Source	Type	Statistical distribution	Sensitivity coefficient	Value	Standard uncertainty
Voltage	B	Rectangular	8.9×10^{-3}	1.0×10^{-5} (V)	5.1×10^{-6}
Current	B	Rectangular	6.1×10^{-3}	1.0×10^{-5} (A)	3.5×10^{-6}
Repeated measurement (current)	A	Normal	8.9×10^{-3}	1.0×10^{-3} (V)	8.9×10^{-6}
Repeated measurement (voltage)	A	Normal	6.1×10^{-3}	1.0×10^{-3} (A)	6.1×10^{-6}

Table S4. Sub-budget for overall uncertainty for conversion efficiency *η*

Component	Combined uncertainty (%)
Input heat flow Q_{in}	0.5
Temperature difference ΔT	0.5
Electrical output P	0.1
Combined uncertainty	0.7
Expanded uncertainty (k = 2)	1.4

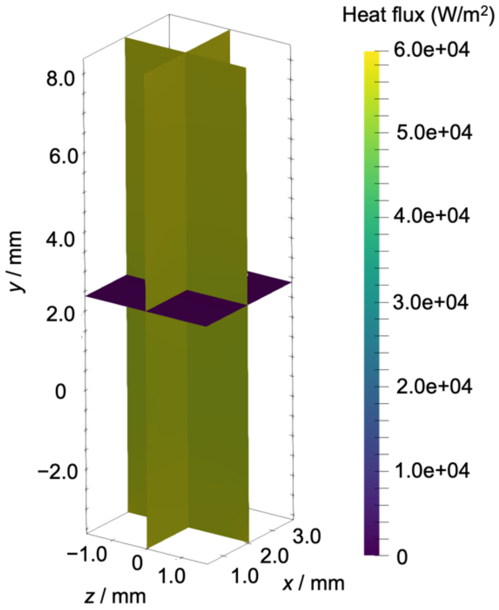

Figure S1. Color maps of heat flux components for a representative thermoelectric leg section. Axial direction: Heat flux distribution shown in the *xy*- and *yz*-planes, illustrating dominant axial conduction. In-plane direction: Heat flux distribution in the *xz*-plane, where radial components are negligible compared to axial flux.

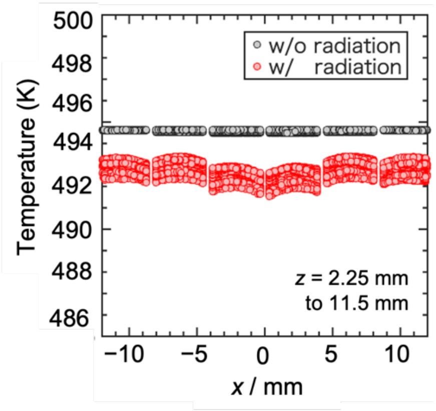

Figure S2. Comparison of leg temperature profiles with and without surface-to-surface radiation. Without radiation: Legs exhibit nearly flat temperature profiles along their height. With radiation: Pronounced concavity (upward convex depression) appears, consistent with Fig. 2(h) in the main text. This effect is attributed to the radiative exchange between adjacent leg sidewalls, which cannot be captured by the conduction-only analysis.
